# New York Medical Jurisprudence Society

**Published:** 1902-01

**Authors:** 


### New York Medical Jurisprudence Society.

*Stated Meeting, December 9, 1901.*

### COMMERCIALISM AND RESPONSIBILITY IN CONNECTION WITH ANTITOXIN AND VACCINE VIRUS.

THE paper of the evening above-named was delivered by Dr. W. R. Inge Dalton in which he criticised the system employed by the Board of Health in the manufacture and distribution of antitoxin sera and vaccine virus. In part he said:

Deplorable as is the loss of life which has occurred in St. Louis, Camden, Cleveland, Atlantic City, and elsewhere from the use of impure antitoxin and from tetanus following vaccination, there is something infinitely more to be regretted, and which gives vastly more cause for alarm, and that is the distrust which has thus been engendered in vaccination and antidiphtheritic serum, two of the greatest life-saving agencies which medical men have at their command.

There is no exaggeration in the statement of one of our leading medical journals, that the deaths in St. Louis sink into positive insignificance compared with the untold thousands of avoidable diphtheria deaths which will inevitably follow, unless members of the medical profession demand a guaranteed purity of antitoxin, and are thus enabled to speak with the confidence of definite knowledge and so inspire the anxious parents with their own confidence.

And in regard to smallpox, the evils to be apprehended are even greater. With what ill-concealed delight will not the unfortunate accidents be made use of by the fanatical opponents of vaccination? What unscrupulous use may we not expect to have made of them by charlatans of every kind?

Upon whom then must the responsibility be placed for these various fatalities? Evidently, on one of three classes,—the manufacturers of the antitoxin and vaccine virus, the physicians, or the parents or guardians of the unfortunate victims.

In regard to the deaths from tetanus following the use of antitoxin at St. Louis, the coroner's jury has spoken with no uncertain voice. Negligence upon the part of the health department in the preparation of the said diphtheria antitoxin and in the issuance thereof is given as the cause of the frightful mortality.

Nor was it one careless act alone, or the mistake of a single individual, that led to the fatalities. The whole system on which the board has been in the habit of producing antitoxin seems to have been unscientific, and in all respects bad.

Far from there being any reason for boards of health engaging in such industries, all considerations of an economical and sociological as well as of a hygienic and scientific character, point to the fact that it would be infinitely better for them to confine themselves to their own duties.

Surely, in this way they would serve a higher purpose than by each of them setting up his own poorly equipped laboratory, and seeking to compete with manufacturing houses, which have millions invested in their plants and which are compelled by a healthy regard for their own interests to adopt every conceivable precaution, to prevent anything but the most perfect material being sent out under their label.

Dr. Dalton then turned to the system in vogue in New York, and cited from the testimony of Dr. John B..Crosby, given before the Mazet committee, at Albany, in 1899, to prove that inferior antitoxin manufactured by the board of health, had been sold to Chicago at reduced rates, and concluded with a vigorous attack upon the sanitary conditions of the board of health stables, where are kept the animals from which the serum is drawn.

Dr. Dalton's paper precipitated a heated discussion, some of the more important points are as follows: Dr. Love, in the course of his remarks, drew particular attention to the potency of certain brands of virus, particularly that of the board of health in producing suppurating wounds which predisposed to more serious septic infection. Alluding to the remarks of some of the speakers, "that the vaccine virus manufactured by a certain reputable pharmaceutical house was not so effective in producing vaccinia as the virus of the board of health," Dr. Love said that the so-called milder virus always produced typical vaccine vesicles in infants, and though the same result was not always obtained after single operation on adults, he thought that true vaccinia followed at least quite as frequently after vaccination with the so-called milder virus, as after the use of the brands which if they produced typical vesicles they obscured the diagnosis of vaccinia, in many instances, by the production of septic wounds.

Dr. Daniel Lewis whose remarks were characterised by a tone of justice and candor, said: It is customary among physicians to neglect their vaccinated patients. If therefore, this episode,—the deaths of persons in Camden, St. Louis and elsewhere from tetanus,—should serve to bring us to our senses, we shall have profited by an occurrence which, in its essence, is ephemeral and inconclusive.

Dr. Lewis did not think it was within the province of boards of health to manufacture vaccine virus or sera for the purpose of selling it, and, as State Health Commissioner, he said that though the state owned an antitoxin plant for experimental purposes they did not prepare any serum for the market.

The city Health Commissioner, Dr. Wm. T. Jenkins came in just as the discussion was closing and at the request of the chairman made a few impromptu remarks in which he discountenanced the sale of sera and virus by the board of health.

